# Ferroelectric phase-transition frustration near a tricritical composition point

**DOI:** 10.1038/s41467-021-25543-1

**Published:** 2021-09-07

**Authors:** Xian-Kui Wei, Sergei Prokhorenko, Bi-Xia Wang, Zenghui Liu, Yu-Juan Xie, Yousra Nahas, Chun-Lin Jia, Rafal E. Dunin-Borkowski, Joachim Mayer, Laurent Bellaiche, Zuo-Guang Ye

**Affiliations:** 1 Ernst Ruska-Centre for Microscopy and Spectroscopy with Electrons, Forschungszentrum Jülich GmbH, Jülich, Germany; 2 Physics Department and Institute for Nanoscience and Engineering, University of Arkansas, Fayetteville, NC USA; 3 Department of Chemistry and 4D LABS, Simon Fraser University, Burnaby, BC Canada; 4 Electronic Materials Research Laboratory, Key Laboratory of the Ministry of Education and International Center for Dielectric Research, Xi’an Jiaotong University, Xi’an, China; 5 School of Microelectronics, Xi’an Jiaotong University, Xi’an, China

**Keywords:** Ferroelectrics and multiferroics, Information storage, Phase transitions and critical phenomena

## Abstract

Phase transition describes a mutational behavior of matter states at a critical transition temperature or external field. Despite the phase-transition orders are well sorted by classic thermodynamic theory, ambiguous situations interposed between the first- and second-order transitions were exposed one after another. Here, we report discovery of phase-transition frustration near a tricritical composition point in ferroelectric Pb(Zr_1-x_Ti_x_)O_3_. Our multi-scale transmission electron microscopy characterization reveals a number of geometrically frustrated microstructure features such as self-assembled hierarchical domain structure, degeneracy of mesoscale domain tetragonality and decoupled polarization-strain relationship. Associated with deviation from the classic mean-field theory, dielectric critical exponent anomalies and temperature dependent birefringence data unveil that the frustrated transition order stems from intricate competition of short-range polar orders and their decoupling to long-range lattice deformation. With supports from effective Hamiltonian Monte Carlo simulations, our findings point out a potentially universal mechanism to comprehend the abnormal critical phenomena occurring in phase-transition materials.

## Abstract (web summary)

Phase transition brings a plethora of exotic phenomena and intriguing effects such as spin and charge frustration. However, the phase transition order is not always explicit. Here, the authors discover phase transition frustration near a tricritical composition point in ferroelectric Pb(Zr,Ti)O_3_.

## Introduction

As a time-honored research topic, phase transition (PhT) covers a broad range of intriguing physical phenomena such as giant electromechanical response^1,2^, magnetoelectric multiferroicity^3^, superconductivity^4^, etc. The classic thermodynamic theory^5^ expounds that the first- and second-order transitions are characteristic of specific signatures such as latent heat, volume change and divergent correlation length. However, an affirmative judgment on order of the transition cannot be made for cases with ambiguous situations^6–9^, which therefore leaves an unfilled gap with the existing theory^10–12^. Geometric frustration depicts an intrinsic incompatibility of some fundamental interactions with respect to the underlying lattice geometry. Stimulated by this, a plethora of unusual phenomena and intriguing effects have been reported, e.g., spin liquids and spin ice^13^, glass-to-crystalline transitions^14^, and exotic spiral ferroelectric states^15,16^. Given their commonality in phenomenological abnormality about PhT, a potential competition between the first- and second-order transitions at the tricritical point of ferroelectric Pb(Zr_1−*x*_Ti_*x*_)O_3_ (PZT, *x* ≥ 0.50) is investigated in this work.

## Results

### Mesoscopic-scale structural anomaly

**Fig. 1:**
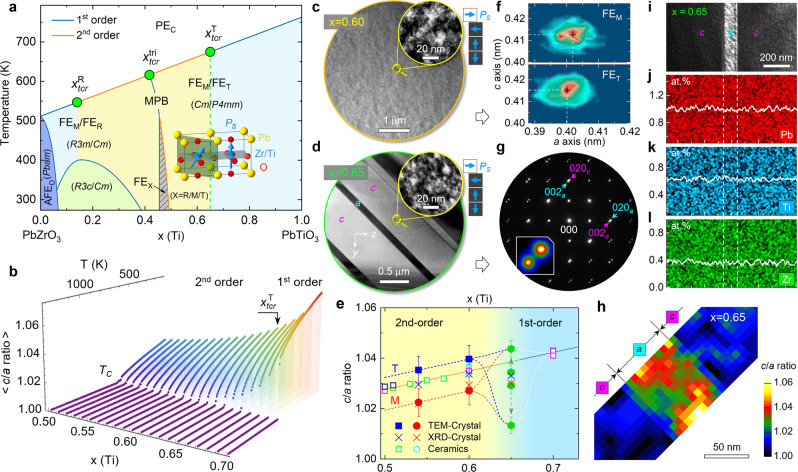
Phase diagram, domain structure, and lattice tetragonality. **a** Schematic phase diagram of PZT (PE_C_—paraelectric, AFE_O_—antiferroelectric, FE_R_—rhombohedral) with annotation of tricritical points (${x}_{\mathrm {tcr}}^{{{{{{\rm{R}}}}}}}$, ${x}_{\mathrm {tcr}}^{{{{{{\rm{tri}}}}}}}$, ${x}_{\mathrm {tcr}}^{{{{{{\rm{T}}}}}}}$: solid green circle) and FE_M_/FE_T_ unit cell. **b** Temperature dependent average *c*/*a* ratio obtained by effective Hamiltonian Monte Carlo simulations with ${x}_{\mathrm {tcr}}^{{{{{{\rm{T}}}}}}}$ ≈ 0.65. **c**, **d** Bright- and dark-field (***g*** = 020_T_) TEM images (inset) of *x* = 0.60 and 0.65 crystals recorded along [100]_T_ zone axis, respectively. **e** Phase tetragonality-composition diagram and postulated traces of FE_T_ (blue) and FE_M_ (red) phases at room temperature. The gray arrows denote changes of ferroelastic domain tetragonality with time at ${x}_{\mathrm {tcr}}^{{{{{{\rm{T}}}}}}}$ and the error bars are s.d. **f** 2D lattice parameter plot for *x* = 0.60 crystal extracted from high-resolution TEM image with (*c*/*a*)_T_ = 1.038 and (*c*/*a*)_M_ = 1.027. **g**–**l** SAED pattern along [100]_T_ direction, representative (020)_*a*_/(002)_*c*_ reflections (inset, log plot), 2D *c/a* ratio map, dark-field image, elemental maps, and line profiles of *x* = 0.65 crystal, respectively. The Zr/Ti molar ratio is measured as Zr:Ti = 37:63.

Experimental and theoretical studies have revealed that there exist three tricritical points in the phase diagram of PZT (Fig. 1a). Given the complex structural evolution on the rhombohedral side (0.06 ≤ ${x}_{\mathrm {tcr}}^{{{\mathrm R}}}$ ≤ 0.26)^17,18^ and at the morphotropic phase boundary (MPB, ${x}_{\mathrm {tcr}}^{{{{{{\rm{tri}}}}}}}$ ≈ 0.45)^12,19,20^, we hereby focus our attention on the tetragonal-side tricritical point, ${x}_{\mathrm {tcr}}^{{{\mathrm T}}}$, which was reported to locate in the 0.6 ≤ ${x}_{\mathrm {tcr}}^{{{{{{\rm{T}}}}}}}$ ≤ 0.7 composition range^12,17,21^. By probing temperature-dependent spontaneous polarization (*P*_S_), dielectric constant (*ε*), and lattice tetragonality (*c*/*a* ratio), a continuous-to-discontinuous transition of the physical quantities at Curie temperature (*T*_C_) evidences that the second-order transition changes to the first-order one at ${x}_{\mathrm {tcr}}^{{{{{{\rm{T}}}}}}}$ ≈ 0.65 with increasing *x* (Fig. 1b and Supplementary Fig. 1). On this basis, the correlation of ferroelectric domain morphology with the PhT order is probed using dark-field transmission electron microscopy (TEM). To distinguish the domain polarization orientation, the failure of Friedel’s law due to dynamical scattering^22^ is considered, i.e., the domains show bright contrast once the ***P***.***g*** > 0 (***P***, a component of ***P***_**S**_; ***g***, scattering vector) criterion is satisfied under two-beam conditions.

We find that the PZT crystals with *x* < ${x}_{\mathrm {tcr}}^{{{{{{\rm{T}}}}}}}$ are characteristic of nesting ferroelectric tetragonal (FE_T_) and monoclinic (FE_M_) domains at nanometer scale (Fig. 1c and Supplementary Fig. 2). In sharp contrast, the *x* = 0.65 crystal is comprised of regularly arranged wide *c* domains (~1 µm) and narrow *a* domains (~100 nm), which is analogous to PbTiO_3_ (refs. ^23,24^) undergoing the first-order transition (Fig. 1d). However, diffraction contrast analysis unveils that the ferroelastic domains are composed of self-assembled FE_T_ and FE_M_ nanodomains (inset in Fig. 1d). Being consistent with the neutron diffraction result^25^ at *x* < ${x}_{\mathrm {tcr}}^{{{{{{\rm{T}}}}}}}$, our effective Hamiltonian Monte Carlo simulations further reveal that the structural phase of first-order-transition PZT is dominated by the FE_T_ phase at *x* > ${x}_{\mathrm {tcr}}^{{{{{{\rm{T}}}}}}}$ (Supplementary Fig. 3). Given the stable coexistence of different phases at temperature *t* < *T*_C_, the unique hierarchical domain configuration observed in ${x}_{\mathrm {tcr}}^{{{{{{\rm{T}}}}}}}$ = 0.65 crystal therefore implies a geometric frustration between the PhT orders.

For the *x* < ${x}_{\mathrm {tcr}}^{{{{{{\rm{T}}}}}}}$ crystals, analysis on X-ray diffraction data shows that lattice tetragonality of the FE_T_ phase is larger than that of the FE_M_ phase, which agrees well with the data of PZT ceramics^25–28^ and is corroborated by our TEM data obtained from local regions (Fig. 1e, f). For the *x* = 0.65 crystal, the *c*/*a* ratio of the tetragonal phase is also suggested to be larger than the monoclinic phase, (*c*/*a*)_T_ = 1.033 and (*c*/*a*)_M_ = 1.030, both of which are smaller than the expected values in the overall trend. However, our selected area electron diffraction (SAED) experiments surprisingly reveal that the two distinct lattice ratios stem from the ferroelastic *a* and *c* domains separately, which are measured as (*c*/*a*)_*a*_ = 1.034 and (*c*/*a*)_*c*_ = 1.029. After month-level storage, the domain lattice ratio is further increased to (*c/a*)_*a*_ = 1.044 ± 0.003 and decreased to (*c/a*)_*c*_ = 1.014 ± 0.003, which is confirmed by real-space mapping using the 4D scanning TEM technique (Fig. 1g, h and Supplementary Fig. 4). The degeneracy of phase tetragonality within mesoscale domains further suggests the scenario of PhT frustration occurred at the ${x}_{\mathrm {tcr}}^{{{{{{\rm{T}}}}}}}$. It is noteworthy that the structural anomaly is irrelevant to compositional segregation, which is evidenced by energy dispersive X-ray spectroscopy maps of the elements (Fig. 1i–l).

### Decoupled polarization–strain relationship

**Fig. 2:**
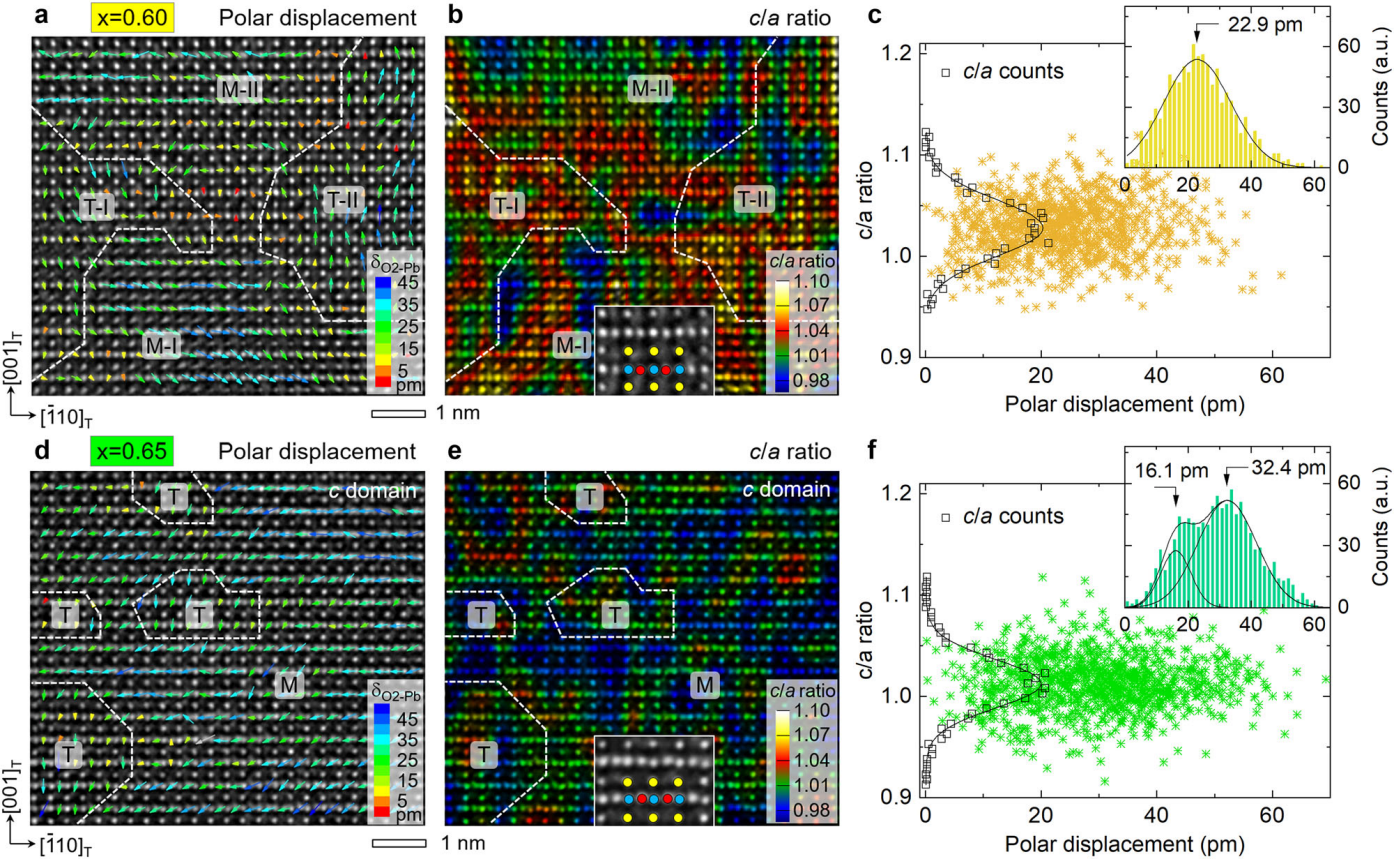
Nanoscale phase coexistence and polarization–strain relation. **a**, **d** Atomic-resolution TEM image of *x* = 0.60 and 0.65 crystal recorded along [110]_T_ direction and overlaid with a map of O_2_ column displacements (color arrows) relative to the centers of Pb/O1 columns (*δ*_O2-Pb_), respectively. **b**, **e** Unit-cell-wise *c/a* ratio map (after interpolation) overlaid on the atomic-resolution TEM image of *x* = 0.60 and 0.65 crystal, respectively. The white dotted lines in (**a**, **b**) and (**d**, **e**) mark the tetragonal (T)/monoclinic (M) phase boundaries. The atomic column types are denoted with Pb/O1—yellow, Zr/Ti—blue, O_2_—red. **c**, **f** Unit-cell-wise correlation of *c*/*a* ratio with O_2_ polar displacement for the *x* = 0.60 and 0.65 crystal, respectively. The interior inset is a statistical profile of the *c*/*a* ratio (squares) fitted by a Gaussian function (solid line) and the right-side inset is a corresponding statistical histogram of the polar displacement.

Our statistical measurement shows that the average domain size is very small, <*d* > ≈ 5.3 nm in the PZT crystals (Supplementary Fig. 5). According to the classic domain theory^5^, *d* ∝ $\sqrt{{E}_{\mathrm {DW}}}$ (*E*_DW_: domain wall energy), this suggests that the *E*_DW_ is very low, which monotonically decreases from ca. 250 to 38 mJ m^−2^, via a concave inflection point at ${x}_{\mathrm {tcr}}^{{{{{{\rm{T}}}}}}}$, with decreasing *x* in terms of our calculation (Supplementary Figs. 6 and 7). Further, atomic-scale domain structures were acquired using the negative spherical-aberration imaging technique^29^. Owing to instability of ordered state to random fields^30^, the nanodomains form irregular configuration in the *x* < ${x}_{\mathrm {tcr}}^{{{{{{\rm{T}}}}}}}$ crystals^31^ and the lattice tetragonality shows obvious fluctuation in real space. This is well manifested by a topological vortex structure, where the average lattice ratio for the FE_T_ and FE_M_ phase is measured as (*c*/*a*)_T_ ≈ 1.036 and (*c*/*a*)_M_ ≈ 1.022 (Fig. 2a, b and Supplementary Fig. 8a–d). A unit-cell-wise correlation of lattice ratio with polar displacement of oxygen (*δ*_O2-Pb_) is plotted for further data statistics and analysis (Fig. 2c). We find that the *c*/*a* ratio and *δ*_O2-Pb_ both follow a Gaussian-type distribution and separately peak at 1.028 (full-width at half-maximum, FWHM = 0.066) and 22.9 pm. Together with individual phase analysis, this evidences a coupled polarization–strain relation, ${P}_{\mathrm S}^{2}=$*σ* (*σ* = *c*/*a* − 1)^32^, in the second-order-transition crystals (Supplementary Fig. 8e, f).

In the *x* = 0.65 crystal, coexisting tetragonal and monoclinic phases and degeneracy of their lattice tetragonality are also confirmed by analysis on atomic-scale TEM images, e.g., the *c*/*a* ratio histogram peaks at 1.012 for the *c* domain and its FWHM is narrowed to 0.054 (Fig. 2d–f). Specifically, we find that the polar displacement of oxygen (*δ*_O2-Pb_) exhibits a bimodal distribution, whose peaks locate around 16.1 and 32.4 pm, respectively. Clearly, this evidences a decoupled *P*_S_ ~ *σ* relation in the tricritical ferroelectric, which is further supported by strongly charged but unstable ferroelastic domain walls if the coupling relation still holds (see “Methods”). On the first-order transition side (*x* > ${x}_{\mathrm {tcr}}^{{{{{{\rm{T}}}}}}}$), the coupled *P*_S_ ~ *σ* relation is verified again by our quantitative measurement on atomically resolved tetragonal ferroelastic domains (Supplementary Fig. 9). These atomic-scale details further indicate a frustrated behavior of PhT at the tricritical point.

### Abnormal critical exponents

**Fig. 3:**
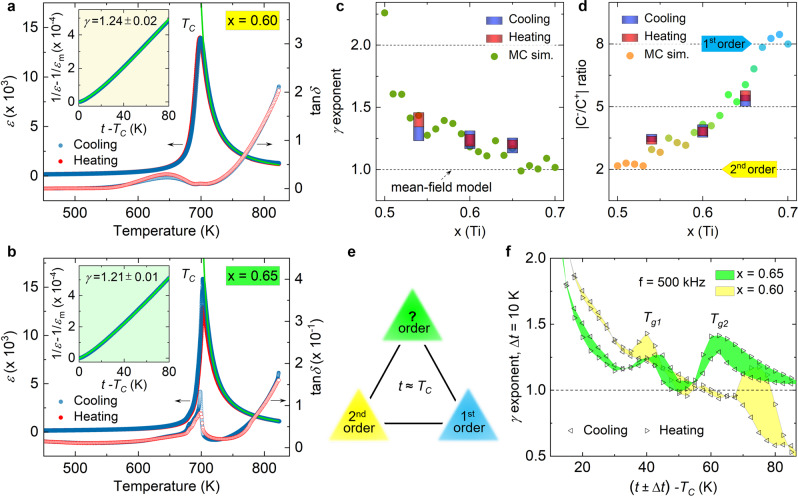
Dielectric constants and critical exponents. **a**, **b** Temperature-dependent *ε* for *x* = 0.60 (*T*_C_ ≈ 699 K) and 0.65 (*T*_C_ ≈ 703 K) crystal measured at *f* = 500 kHz, respectively. The green solid lines are fitting to the cooling *ε* data at *t* > *T*_C_ using a modified Curie–Weiss law 1/*ε* − 1/*ε*_*m*_ = *(t* − *T*_0_)^*γ*^/*C*. **c**, **d** Evolution of *γ* exponent and Curie-constant ratio |*C*^−^/*C*^+^| from effective Hamiltonian Monte Carlo simulations and their experimental values measured at *f* = 500, 333, and 100 kHz, respectively. **e** Illustration of the frustrated PhT order, with |*C*^−^/*C*^+^| ≈ 5 and *γ* > 1, arising from competing first- and second-order transitions at ${x}_{\mathrm {tcr}}^{{{{{{\rm{T}}}}}}}$ and *T*_C_. **f** Temperature-dependent *γ* exponent measured within a restricted temperature interval (Δ*t* = 10 K) at *t* > *T*_C_ and *f* = 500 kHz for *x* = 0.65 and 0.60 crystal (see Supplementary Fig. 11 for *x* = 0.54 case).

To verify the frustration scenario at the ${x}_{\mathrm {tcr}}^{{{{{{\rm{T}}}}}}}$, critical exponents were analyzed from temperature dependent dielectric constant (*ε*) of the PZT crystals (Fig. 3a, b and Supplementary Fig. 10). By fitting *ε* at *t* > *T*_C_ using a modified Curie–Weiss law, 1/*ε* − 1/*ε*_*m*_ = (*t* − *T*_*0*_)^*γ*^/*C* (*ε*_*m*_: the maximum of *ε*, *C*: Curie constant, *T*_0_: Curie–Weiss temperature), we find that the *x* ≤ ${x}_{\mathrm {tcr}}^{{{{{{\rm{T}}}}}}}$ PZT crystals are characteristic of a pronounced precursor behavior^33^. This is manifested by a large gamma exponent (*γ* > 1) and deviation from the Curie–Weiss law due to formation of polar clusters at *t* > *T*_C_^6,7,15^. A systematic change of *γ* is unveiled by our effective Hamiltonian Monte Carlo simulations, which decreases from 2.26 to 1.17 with increasing *x* at *x* ≤ ${x}_{\mathrm {tcr}}^{{{{{{\rm{T}}}}}}}$, above which the classic mean-field value (*γ* = 1) is observed (Fig. 3c). In addition to this, Curie-constant ratio (|*C*^−^/*C*^+^|) is measured by fitting *ε* below (*C*^−^) and above (*C*^+^) *T*_C_ using Curie-Weiss law to testify the PhT orders according to the Landau–Devonshire theory^5^. Associated with a step-wise increase from 2 (second order) to 8 (first order) with *x*, the |*C*^−^/*C*^+^|ratios of *x* = 0.54 and 0.60 crystals are found to be 3.45 ± 0.19 and 3.83 ± 0.26, respectively. This ratio is measured as |*C*^−^/*C*^+^| ≈ 5.4 for the *x* = 0.65 crystal (Fig. 3d), which differs from the mean-field value of 4 at the tricritical point^34^.

The critical exponent anomalies verify the existence of a frustrated PhT order at the ${x}_{\mathrm {tcr}}^{{{{{{\rm{T}}}}}}}$ (Fig. 3e). Given the nearly coincident loss tangent (tan*δ*) of the *x* < ${x}_{\mathrm {tcr}}^{{{{{{\rm{T}}}}}}}$ crystals, the discernible thermal hysteresis at *T*_C_ of x = 0.65 crystal further hints an anomaly of the PhT (Fig. 3a, b). To find out origin of the frustration, the precursor dynamics reflecting optic-acoustic mode-mode coupling at *t* > *T*_C_ was probed by analyzing local *γ* exponent within a restricted temperature interval (Δ*t* = 10 K)^35^. For the *x* = 0.65 crystal, two localized polar glassy states featured by gamma (*γ*) peaks were identified at *T*_*g1*_ = *t* − *T*_C_ ≈ 40 K and *T*_*g2*_ = *t* − *T*_*C*_ ≈ 63 K, respectively (Fig. 3f and Supplementary Fig. 11). Around *T*_*g1*_, the gamma has a peak value of *γ* ≈ 1.25 in both heating- and cooling-cycle profiles, while the peak value at *T*_*g2*_ decreases from ~1.46 in the heating cycle to ~1.30 in the cooling cycle. Critical behavior study indicates that corresponding to the characteristic *γ* value, the polar glass states adopt short-range 3D-Ising (*γ* ≈ 1.25) and long-range 3D-random-Ising (*γ* ≈ 1.46) universality classes^6^, respectively. This reveals that accompanied with heating- and cooling-dependent structural changes, the frustrated PhT order may result from an intricate interplay of competing short-range dipolar orders with spontaneously developed long-range ones^36^ around *t* *T*_C_. Despite the *γ* exponent being large (1.22 ≤ *γ* ≤ 1.44) in the *x* < ${x}_{\mathrm {tcr}}^{{{{{{\rm{T}}}}}}}$ crystals as well, a similar competition is not established due to presence of single *γ* peak around either *T*_*g1*_ or *T*_*g2*_ in the heating or cooling cycle. Additionally, we also notice that the tan*δ* in *x* < ${x}_{\mathrm {tcr}}^{{{{{{\rm{T}}}}}}}$ crystals is one order of magnitude higher than that in the *x* = 0.65 crystal, which can be attributed to the formation of conductive channels due to random arrangement of nanodomains^31,37,38^.

### Birefringent evidence of PhT frustration

**Fig. 4:**
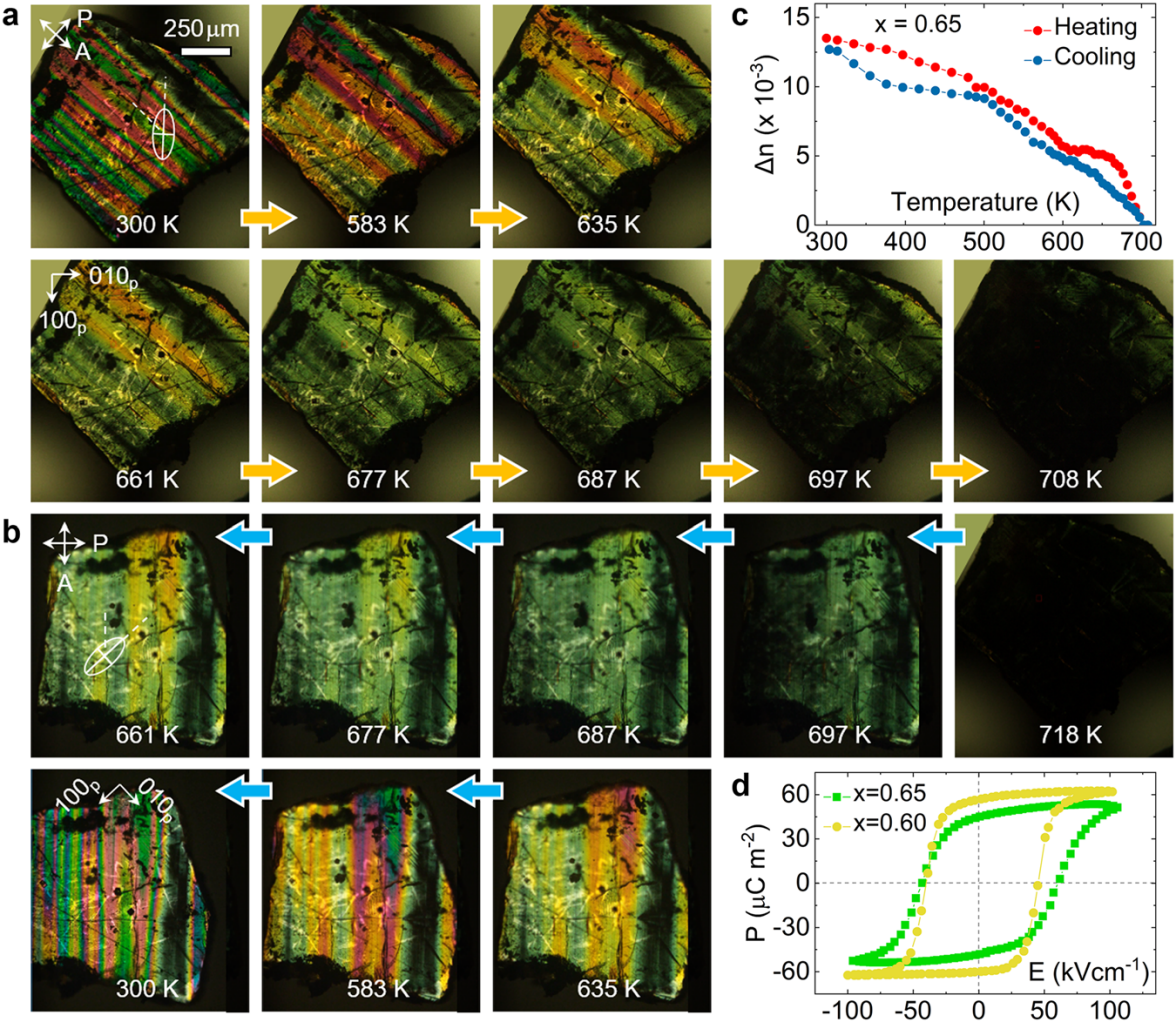
Evidence of competing first- and second-order transitions at ${{x}}_{{\mathrm {tcr}}}^{\mathrm {T}}$ around *T*_C_ and ferroelectric anomaly. **a**, **b** Temperature-dependent ferroelastic domain structure evolution in a pseudocubic (001)_p_ platelet of the *x* = 0.65 PZT crystal observed during heating and cooling cycles by PLM, respectively. The polarizer (P), analyzer (A), and optical indicatrix are denoted on the image with respect to the crystallographic orientation. **c** Temperature-dependent birefringence data of the *x* = 0.65 PZT crystal. **d** *P–E* hysteresis loops of the *x* = 0.60 and 0.65 PZT crystals measured at room temperature with frequency *f*  = 10 Hz.

Being consistent with the precursor dynamics identified at *t* > *T*_C_, our variable-temperature polarized light microscopy (PLM) experiments directly reveal the competion of PhT orders at *t* ≤ *T*_C_. From heating-cycle PLM snapshots, we see that the mesoscale band-like ferroelastic domain array is well preserved until *t* ≈ *T*_C_, at which the domain width and wall position start to evolve dynamically (Fig. 4a). Preservation of the long-range dipolar correlation indicates that the first-order transition overbears the second-order one^39^, which is evidenced by an abrupt drop of birefringence (Δ*n*) as *t* approaching *T*_C_ (Fig. 4c). However, the formation of band-like ferroelastic domains is much delayed in the cooling cycle (Fig. 4b), which can be identified from the domain-contrast change at the identical *t* points ranging from 697 to 635 K. The enhanced short-range dipolar correlation suggests that the second-order transition prevails over the first-order one^40^, as proved by a smooth transition of Δ*n* around *T*_C_ (Fig. 4c). On the physical property aspect, the PhT frustration is also manifested by abnormal ferroelectric property at room temperature (Fig. 4d). With respect to the symmetric polarization–electric field (*P–E*) loops of PZT undergoing the first-^41^ and second-order transitions, we find that the remnant polarization is reduced by ~20.4% relative to the *x* < ${x}_{\mathrm {tcr}}^{{{{{{\rm{T}}}}}}}$ PZT crystals. Furthermore, a very large built-in field (Δ*E*_C_ ≈ 20 kV cm^−1^), an order of magnitude higher than that of *x* < ${x}_{\mathrm {tcr}}^{{{{{{\rm{T}}}}}}}$ crystals, is observed in the tricritical ferroelectric.

### Monte Carlo simulations

**Fig. 5:**
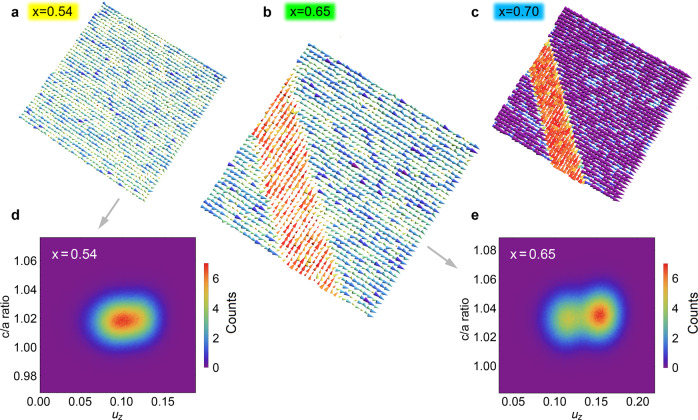
Monte Carlo simulated domain structure evolution near ${{x}}_{{\mathrm {tcr}}}^{\mathrm T}$ at room temperature. **a** Nesting tetragonal and monoclinic nanodomains induced by second-order transition in *x* < ${x}_{\mathrm {tcr}}^{{{{{{\rm{T}}}}}}}$ PZT. **b** Hierarchical domain structure induced by frustrated-order transition in *x* = ${x}_{\mathrm {tcr}}^{{{{{{\rm{T}}}}}}}$ PZT. **c** Ferroelastic 90° domain structure induced by first-order transition in *x* > ${x}_{tcr}^{{{{{{\rm{T}}}}}}}$ PZT. The local electric dipoles are colored according to their projection on the [010]_p_ direction while the arrowhead size indicates the magnitude of the electric dipole. The supercells (40 × 40 × 5 pseudocubic unit cells) are viewed along the [001]_p_ direction. **d**, **e** Joint probability distribution of local mode (*u*_*z*_) and local tetragonality for **d** *x* = 0.54 and **e** either *c* or *a* domain of *x* = 0.65 PZT supercells, respectively. The colors denote probability distribution in the 2D correlation map.

To further verify the experimental results, we performed effective Hamiltonian Monte Carlo simulations on 80 × 80 × 5 supercells of PZT near ${x}_{\mathrm {tcr}}^{{{{{{\rm{T}}}}}}}$ at room temperature, in which only tetragonal ferroelastic *c* and *a* domains with equivalent volume, identical dipolar magnitude, and orientation along [001]_T_ are configured at the initial state. After relaxation for 40,000 Monte Carlo sweeps, we find that the initial *c* and *a* domains in the second-order-transition PZT (*x* < ${x}_{\mathrm {tcr}}^{{{{{{\rm{T}}}}}}}$) supercells disappear and the entire structure transforms into coexisting tetragonal or monoclinic nanodomains, whose characteristic size is several nanometers (Fig. 5a). In good agreement with the experimental result, the hierarchical domain structure is nicely reproduced in frustrated-order-transition PZT supercells with the Ti concentration around ${x}_{\mathrm {tcr}}^{{{{{{\rm{T}}}}}}}$. Meanwhile, the nanodomain sizes are found to increase gradually with decreasing *x* owing to enhanced flexibility in the orientation of local dipoles (Fig. 5b). On the first-order-transition (*x* > ${x}_{\mathrm {tcr}}^{{{{{{\rm{T}}}}}}}$) side, the ferroelastic 90° domains are preserved due to dominance of the tetragonal phase, albeit with unequal width for the *c* and *a* domains (Fig. 5c). In addition, the *P*_S_ ~ *σ* coupling and decoupling behaviors across the ${x}_{\mathrm {tcr}}^{{{{{{\rm{T}}}}}}}$ are also confirmed in our Monte Carlo simulations (Fig. 5d, e and Supplementary Fig. 12).

### Discussion

Besides the evidences of the critical exponent and birefringence data, the PhT frustration at ${x}_{\mathrm {tcr}}^{{{{{{\rm{T}}}}}}}$ can also be understood from the lattice distortion point of view^36^. With respect to the PE_C_ phase, freezing of the glassy states at *t* < *T*_C_ gives rise to locally favored tetragonal and monoclinic ferroelectric orders, which couple with their individual lattice symmetry^31^. However, as being subject to the long-range lattice deformation, the short-range polar orders compete within the mesoscale domains and fail to simultaneously couple with the uniformly distorted lattices. This leads to geometrically frustrated structural features, e.g., the decoupled *P*_S_ ~ *σ* relation, which can be attributed to the “slower breathing” of dipolar inhomogeneity according to first-principle-based simulations^42^. Accordingly, the greatly diminished polarization anisotropy (see Supplementary Figs. 6 and 7) becomes responsible for the configurational change of ferroelastic domains under varied boundary conditions^12^. Therefore, analogous to the spin and charge frustration observed in ferroic materials^13,43^, the PhT frustration gives rise to an ambiguous PhT order at the tricritical composition point. It is noteworthy that the frustrated transition order is distinct from the coexistence or mixture of first order with second-order transitions^44^, which can easily be identified from the correlation of PhT order with the corresponding structural feature.

Regarding the critical exponent *γ* that is larger than one, it reflects the effective dimensionality of the order parameter and the interactions at play, rather than any disorder or frustration. To verify the existence of geometric frustration, incompatible fundamental interplay of a physical quantity with the lattice geometry and ground state degeneracy should be fulfilled simultaneously^6,13,16,43^. In our Monte Carlo simulations, we find that the tricritical transition takes place in a composition interval of Δ${x}_{\mathrm {tcr}}^{{{{{{\rm{T}}}}}}}$ ≈ 0.10, which is denoted by dashed lines in Fig. 1e. For ferroic and superconducting systems with apparent tricritical points^4,8,34,45^, one may foresee that the PhT frustration occurs as the tricritical point is accessed by tuning composition, electric, magnetic field, and pressure. For systems with hidden tricritical points, e.g., BaTiO_3_, the frustration scenario probably validates as well given the simultaneous presence of critical exponent anomaly (*γ* ≈ 1.08)^7^, structural hierarchy and degenerate lattice ratio for the coexisting tetragonal and monoclinic phases^46,47^. This even applies to the thermotropic phase boundary^47^, where the first-order ferroelectric transition competes with the excited second-order transition, which is evidenced by presence of low-symmetric structural phases^48^.

In summary, we report a frustrated PhT order at the tricritical point of ferroelectric PZT solid solution. Given the ubiquitous tricritical point, our findings suggest a generic mechanism to decipher PhT-related unusual critical phenomena in ferroic materials, which are featured by abnormal critical exponents, structural hierarchy, degenerate lattice tetragonality for coexisting phases, and may possibly be extended to ferroelectrics with diffuse PhTs near the MPBs^7,49^. Particularly, the frustrated PhT scenario offers an important degree of freedom to engineer hierarchical domains, which has been reported to play important roles in improving material performances such as piezoelectricity^50^, magnetoelectric effect^3^, shape memory^51^, and electrostatic energy storage^52^. Therefore, it is believed that this work may inspire extensive research interest on exploring PhT-related frustrated states and design of material functionality in a more flexible way.

## Methods

### Materials preparation

Lead zirconate titanate PZT (*x* = 0.54, 0.60, and 0.65) single crystals were grown by a top-seeded solution growth technique^53^, and the PZT (*x* = 0.90) thin films were grown by pulsed laser deposition^54,55^. PLM (Olympus BX60) was used to characterize temperature-dependent domain evolution data. FEI Helios NanoLab 400s focused ion beam (FIB) system was used for preparing the lamella specimens, before which an Au layer (thickness ~25 nm) was coated on the sample surfaces. To protect the samples from being damaged by Ga ions, electron-induced carbon (~180 nm) and ion-induced Pt (~4 μm) layers were deposited on the region of interests (area ~20 µm × 2 µm). After taking out the lamella by making trenches on both sides, the lamella was welded to the TEM grids, milled at 30 kV with 2.8 nA–93 pA currents and followed by a final cleaning at 5 kV and 47 pA. To remove the surface contamination and damage layer, the NanoMill Model 1040 system operated at 500 V was used to further clean and thin down the lamella specimens.

### Electron microscopy imaging experiment

The dark-field imaging and SAED experiments were performed on an FEI Tecnai F20 microscope operated at 200 kV. Referring to the SrTiO_3_ standard, the lattice ratios of PZT crystals were measured in a quantitative way from the SAED patterns. The atomic-resolution TEM and 4D-STEM experiments were performed on image- and probe-corrected FEI Titan 80-300 microscopes operated at 300 kV, respectively. By fitting atomic column peak intensities using 2D-Gaussian-function-based maximum likelihood estimation^56,57^, we simulated the atomic-resolution images using CrystalKit-MacTempas software package. An FEI Titan 80-200 ChemiSTEM microscope, equipped with a Super-X energy dispersive X-ray spectrometer, was used for compositional analysis.

### Estimation of bound charges at ferroelectric domain wall

Supposing the ${P}_{\mathrm S}^{2}=$*σ* relation holds in the *x* = 0.65 crystal, referring to PbTiO_3_ standard^54^ (*P*_S_ = 96.8 μC cm^−2^, *c*/*a* = 1.0643), the coupling coefficient is determined as *к* = 145,727 μC^2^ cm^−4^. For the ferroelastic domains with (*c*/*a*)_*a*_ = 1.044 and (*c*/*a*)_*c*_ = 1.014, our calculation reveals that the density of bound charges is Δ*P*_S⊥_ = 24.7 μC cm^−2^ normal to the ferroelastic wall plane, which is equal to the *P*_S_ of BaTiO_3_^58^. To lower energy of the strongly charged wall structures, an especially high density of free carriers, beyond the available limit of the material itself^59^, is needed. To minimize the electrostatic energy, the charged domain walls usually show curved morphology^60^. This is also different from our experimental observations. These results therefore alternatively refute the *P*_S_ ~ *σ* coupling relation in the *x* = 0.65 crystal.

### Dielectric and ferroelectric property measurement

The dielectric properties of PZT single crystals were measured using a Novocontrol Alpha high-resolution broadband dielectric spectrometer. The dimensions of the crystals, oriented along (001)_p_, (011)_p,_ and (001)_p_, used for the measurement are 1.4 × 0.8 × 0.162, 1.5 × 1.67 × 0.05, and 1.9 × 1.2 × 0.22 mm^3^ for the *x* = 0.54, 0.60, and 0.65 crystal, respectively. Typically, a small signal ac electric field (1 *V*_rms_) was applied for the standard dielectric spectroscopy measurements. The Curie-constant ratio was measured beside *T*_C_ in a temperature interval of 10–20 K. A standardized ferroelectric analyzer system (TF Analyzer 2000; aixACCT, Germany) was used to measure the ferroelectric property at room temperature.

### Monte Carlo simulations

Monte Carlo simulations on PZT bulks were performed using an ab initio-based effective Hamiltonian model^61,62^, which parametrizes the Born–Oppenheimer energy landscape in terms of local modes about B-site polar displacement, oxygen octahedral rotation, and takes into account acoustic phonon branches parameterized through homogeneous strain tensor and A-site displacement within each unit cell. The alloying effects are mimicked by introducing local fields through breaking local cubic symmetry in the PE state via compositional disorder and different values of on-site coefficients of the effective Hamiltonian. The supercell lateral sizes were chosen to be of 12 × 12 × 12 or 80 × 80 × 5 unit cells along the pseudocubic (p) [100]_p_, [010]_p_, and [001]_p_ axis, respectively. The former and latter geometry was used to obtain temperature-dependent macroscopic property and domain structure, respectively. To compute equilibrium property for each considered composition, we firstly performed temperature annealing simulations to obtain the raw values of *T*_C_, where the system was cooled from 1500 K down to 100 K with a step size of 50 K. For *x* = 54, 60, 65, and 100, the annealing simulations were then repeated under hydrostatic pressure varying from −5 to 5 GPa with an increment of 0.1 GPa. This helps to define the effective pressure and to calibrate the simulated *T*_C_ with respect to the experimental one. By interpolating the effective pressure values, we obtain the external pressure for all considered compositions in the range of *x* = 0.50–0.70. This procedure allows to correct for errors induced by LDA approximation while constructing the effective Hamiltonian model, and the errors related to the absence of anharmonic elastic contributions that are responsible for thermal lattice expansion.

## Supplementary information

Supplementary Information

## Data Availability

The authors declare that all data supporting the findings of this study are available within the paper and its Supplementary information files.

## Code availability

The codes used in this study are available from the corresponding authors upon reasonable request.
