# Supplementary material for: Ferroelectric phase-transition frustration near a tricritical composition point
Source: Nat Commun. 2021 Sep 7;12:5322. doi: 10.1038/s41467-021-25543-1 (PMC8423788; doi:10.1038/s41467-021-25543-1)
Supplement: Supplementary file 1 — Supplementary Information [file 41467_2021_25543_MOESM1_ESM.pdf]

Supplementary Information for

Ferroelectric phase-transition frustration near a tricritical composition point

Xian-Kui Wei[1,6], Sergei Prokhorenko[2,6], Bi-Xia Wang[3], Zenghui Liu[4], Yu-Juan Xie[3], Yousra Nahas[2], Chun-Lin Jia[1,5], Rafal E. Dunin-Borkowski[1], Joachim Mayer[1], Laurent Bellaiche[2]* & Zuo-Guang Ye[3]**

[1]Ernst Ruska-Centre for Microscopy and Spectroscopy with Electrons, Forschungszentrum Jülich GmbH, 52425 Jülich, Germany

[2]Physics Department and Institute for Nanoscience and Engineering, University of Arkansas, Fayetteville 72701, USA

[3]Department of Chemistry and 4D LABS, Simon Fraser University, 8888 University Drive, Burnaby, British Columbia, V5A 1S6, Canada

[4]Electronic Materials Research Laboratory, Key Laboratory of the Ministry of Education and International Center for Dielectric Research, Xi'an Jiaotong University, Xi'an 710049, China

[5]School of Microelectronics, Xi'an Jiaotong University, Xi'an 710049, China

[6]These authors contribute equally to this work: Xian-Kui Wei, Sergei Prokhorenko.

*Correspondence authors. E-mail: x.wei@fz-juelich.de (X.-K.W.); laurent@uark.edu (L.B.); zye@sfu.ca (Z.-G.Y.)

Supplementary discussion

In our study, Monte Carlo simulations were performed to identify location of the tricritical point on the Ti-rich side of PZT. Referring to the experimental T_C values, the simulated ones are calibrated by employing appropriate value of effective hydrostatic pressure in the simulations (Supplementary Fig. 1a,b and Fig. 1b). As a function of Ti content, kinks at T_C are observed at x > 0.65 PZT in the polarization, inverse dielectric constant and average *c*/*a* ratio profiles. This reveals that associated with the continuous-to-discontinuous transition of these physical quantities, the second- to first-order transition takes place at x_{tcr}^{T}= 0.65 in the PZT solution. The result well agrees with the specific heat measurement on the tricritical point[1]. From the inverse dielectric constant, one can see that the x dependent Curie-constant ratio is mainly governed by C^- below T_C, above which C^+ is nearly a constant.

Supplementary Fig. 2a-g shows the domain features of x = 0.54 PZT crystal. Similar to the x = 0.60 crystal, only nanoscale domains are present. The SAED pattern shows that the diffraction spots are usually splitting owing to the coexisting monoclinic and tetragonal phases with different lattice parameters, $(c/a)_\mathrm{T} \approx 1.035$ and $(c/a)_\mathrm{M} \approx 1.022$. This is consistent with 2D lattice parameter plot extracted from a high-resolution TEM image, where the local lattice tetragonality are measured as $(c/a)_\mathrm{T} = 1.030$ and $(c/a)_\mathrm{M} = 1.020$. It should be noted that the lattice tetragonality of the monoclinic phase is defined by referring to that of the tetragonal phase, i.e., $(c/a)_\mathrm{M} = c_\mathrm{T}/a_\mathrm{T}$, where the lattice parameter relations are $a_\mathrm{M} \approx b_\mathrm{M} \approx \sqrt{2}a_\mathrm{T}$ and $c_\mathrm{M} \approx c_\mathrm{T}$. The elemental maps show that the composition is homogeneously distributed in the crystal.

With consideration of oxygen octahedral rotations and multiple local interactions, the temperature dependent phase structures in PZT are clarified in our Monte Carlo simulations (Supplementary Fig. 3a-d). For x below the tricritical composition point, x ≤ 0.65, our simulations show that locally inhomogeneous multiple phases and nanodomains coexist in a wide range of temperatures below T_C. As the Ti content approaches 0.70, low-symmetric monoclinic phases only takes a very small fraction below T_C and the phase structure is governed by the tetragonal phase. The structural phase evolution presented here is consistent with our variable-temperature XRD characterization result on PZT (x = 0.54, 0.60, 0.65 and 0.70) ceramics. This suggests that the multiple phase coexistence facilitates ground-state degeneracy below T_C.

Supplementary Fig. 4a-d shows the configurational sensitivity of tricritical ferroelectric to change of physical boundary conditions. From the morphological comparison, before and after (ca. six-month) storage in vacuum desiccator, we cannot see evident ferroelastic domain change apart from the probable change of surface roughness, which is judged from variation of the image contrast. Our measurement on the SAED patterns reveals that the domain tetragonality is $(c/a)_a \approx 1.034$ and $(c/a)_c \approx 1.029$ in the as-prepared specimen, which evolves to 1.044 and 1.014 after the storage, respectively. In particular, degeneracy of tetragonal and monoclinic phase tetragonality within each ferroelastic domain is verified by Gaussian-type intensity distribution of the diffraction spots. Similar degenerate feature is also identified from $[110]_T$-oriented specimen (Supplementary Fig. 4e,f). The result implies that the high sensitivity of domain tetragonality is probably attributed to surface tension and inherent structural defects[2,3].

From the two-beam dark-field TEM images, we measured and analyzed the size of nanodomains in the PZT crystals (Supplementary Fig. 5a-f). One can see that the domain diameter, in the range of 2 ~ 15 nm, statistically peaks around 5.3 nm for the x = 0.54, 0.60 and 0.65 crystals. On the basis of iterative comparison with the experimental image, our multislice-based quantitative image simulation to a representative region reveals the specimen thickness, used for atomic-scale NSCI imaging, is about 4.6 nm (Supplementary Fig. 5g). This indicates that along the viewing direction, the overlapping between different structural phases is unavoidable at certain places, e.g., the phase boundaries, as the domain size is smaller than the specimen thickness.

In order to characterize the energetics of nanodomain boundaries, we have computed the dependence of the free energy F_θ on the angle θ between the nearest neighbor dipoles at room temperature. Technically F_θ is computed using the histogram approach similar to the method described elsewhere[4]. Namely, we perform Monte Carlo simulations at room temperature initialized from a random dipolar structure. After the convergence is reached, we compute the histogram $H_{\theta,s}$ of angles $\theta_{i,j}$ formed by electric dipoles at the nearest-neighbor sites i and j at each Monte Carlo sweep s. The F_θ function is then approximated as $-k_B T log\langle H_{\theta,s}\rangle_s$, where $\langle\ldots\rangle_s$ denotes the averaging over Monte Carlo sweeps. In the limit of infinite number of sweeps $s \to \infty$, the F_θ is given by

$$F_\theta = -k_B T \; log \int du_i du_j \int \prod_{s \neq i,j} d\boldsymbol{u}_s \, e^{-\beta H},$$

where k_BT denotes the temperature in energy units, Z is the partition function, and the inner integral is carried out over all possible values of local dipoles $\boldsymbol{u}_s$ at sites s of the supercell excluding the two nearest-neighbor sites i and j. For the latter sites, the integration is carried only over the dipole magnitudes $u_i = |\boldsymbol{u}_i|$ and $u_j = |\boldsymbol{u}_j|$. Omitting the integration over the angles for sites i and j makes the integral functionally depend on the angle θ between $\boldsymbol{u}_i$ and $\boldsymbol{u}_j$ and F_θ acquires the meaning of the free-energy-like quantity[5]. The calculated F_θ curves for different compositions are presented in Supplementary Fig. 6a,b.

It can be seen from Supplementary Fig. 6a that at high Ti concentration ($x > x_{tcr}^{\mathrm{T}}$), F_θ features three special points – two minima (labelled 1 and 3) and an inflection point (labelled 2). By definition, the minima of F_θ corresponds to high probability angles between nearest-neighbor dipoles (stable and meta-stable states). Here, the minima (1) and (2) correspond to the equilibrium angles between nearest-neighbor dipoles in the tetragonal domains and at 180° domain walls, respectively. The deviation of the highest minima position from the value of 180° is indicative of the non-zero domain wall width. The inflection point (2) is a relic of 90° domain walls. As discussed previously[6], despite being energetically preferable to 180° domain boundaries, the 90° walls in $PbTiO_3$ (and, likewise, in high-Ti PZT crystals) ought to be highly mobile. Owing to a low barrier for the 90° domain wall motion, the corresponding minima in F_θ becomes smeared and results in an inflection that can be seen in Supplementary Fig. 6b for x = 0.70.

Notably, decreasing the Ti concentration progressively smoothens the F_θ behavior around point (2) and at x=0.65, the F_θ dependence on the θ for 50°-100° range becomes linear (Supplementary Fig. 6b for x=0.65). Similarly, the free-energy cost of 180° relative orientations of nearest neighbor dipoles increasing with decreasing x. These observations hint to increasing mobility of the 90° and 180° domain walls with decreasing Ti concentration. Interestingly, it is important to note that the left-most minima (1) shifts to higher angles and becomes progressively wider when x is decreased. This is a direct evidence of enhanced orientational flexibility of local dipoles and a decreasing free-energy cost of nano-domain boundaries.

To further probe the energetics of domain wall boundaries, we have computed the energy difference between the 90° domain states (pertinent to Fig. 5) and mesoscopically homogenous polar configurations. The dependence of the resulting 90° domain wall energy density is shown below. Concordantly with the conclusion obtained from F_θ analysis, we see a decreasing energy

density with decreasing x. At the same time, we would like to make a cautionary note - the obtained energy densities are higher than the estimates from first-principles calculations[6]. Such mismatch is due to the employed effective Hamiltonian model approximations (e.g. not all relevant degrees of freedom taken into account). Because of this, the results should be considered only at a qualitative comparison level (Supplementary Fig. 7).

Through analyzing the atomic-resolution TEM images, obvious spatial fluctuation in c/a ratio of tetragonal and monoclinic phases is also observed in the x = 0.54 crystal (Supplementary Fig. 8a-d). The unimodal distribution of c/a ratio and polar displacement $\delta_{\text{O2-Pb}}$ in the unit-cell-wise correlation map also evidences the polarization-strain coupling relationship. For PZT crystals undergoing the second-order transition, we also analyze the relationship of c/a ratio with $\delta_{\text{O2-Pb}}$ in the tetragonal and monoclinic phases (Supplementary Fig. 8e,f). The statistical results obtained from Fig. 2a show that the tetragonal domains with larger c/a ratio (1.036) are characteristic of larger polar displacement ($\delta_{\text{O2-Pb}}$ = 24.7 pm), while the monoclinic domains with smaller c/a ratio (1.022) correspond to smaller polar displacement ($\delta_{\text{O2-Pb}}$ = 21.3 pm).

Analogously, based on quantitative measurement on atomic positions, our analysis reveals that the $P_S \sim \sigma$ coupling relation holds again in the x = 0.90 PZT (Supplementary Fig. 9a-d). The statistical histograms show that the c/a ratio and $\delta_{\text{O2-Pb}}$ is peaked at 1.063 and 27.3 pm, respectively. By using annular-bright-field (ABF) imaging technique of scanning TEM, the correlation of c/a ratio with polar displacement of Zr/Ti columns, relative to centers of nearest-neighboring Pb columns, is examined for a pair of adjacent c and a domains in this sample (Supplementary Fig. 9e,f). The result shows that the relation chart well overlaps with each other between these two domains, where their c/a ratio and $\delta_{\text{Zr/Ti-Pb}}$ are peaked around 1.059 and 14.2 pm, respectively.

Supplementary Fig. 10a-c show the temperature dependent dielectric constant and loss tangent of x = 0.54, 0.60 and 0.65 crystal, their Curie temperature is T_C = 682.3 K, 699.5 K and 702.7 K, respectively. The loss tangent profiles show that thermal hysteresis near T_C is absent in the second-order-transition crystals. With reduction of $\tan\delta$ by one order of magnitude, the thermal hysteresis is clearly visible in the x_{tcr}^{T} crystal. Supplementary Fig. 10d-g show the Monte Carlo simulated $1/\varepsilon$ profiles and their fit using the modified Curie-Weiss law. One can see the evolution of γ exponent pertinent to the frustration behavior[7,8] near x_{tcr}^{T} at $t > T_C$ temperature region.

Supplementary Fig. 11a presents the frequency dependent precursor effect for the x_{tcr}^{T} = 0.65 crystal. With lowering of the test frequency, we see that the dynamic γ exponent is decreasing from 1.27 to 1.17 at the T_{g1}. At the T_{g2}, the gamma peak is decomposed due to enhancement of the loss tangent. This is consistent with the fact that the long-range electrostatic force is vulnerable to *dc* conductivity in ordinary ferroelectrics[9,10]. In the x = 0.54 crystal, only one γ peak, with strong fluctuation between 1.5 and 0.5, is observed at either T_{g1} or T_{g2} (Supplementary Fig. 11b). By fitting the $1/\varepsilon$ data with varied starting temperature away from T_C, we see that the γ exponent essentially keeps a constant value in the x_{tcr}^{T} = 0.65 crystal. However, the γ exponent rapidly decreases to the mean-field value of 1.0 with increase of Δt (Supplementary Fig. 11c). This further confirms the interplay of competing dipolar orders with long-range lattice deformation occurred in the tricritical ferroelectric as temperature lowers to $t < T_C$.

Corresponding to the coexisting nanodomains (Fig. 5a), analysis on the c/a ratio shows that $(c/a)_\mathrm{M}$ < $(c/a)_\mathrm{T}$ in the second-order-transition PZT, which qualitatively agrees with the experimental results (Supplementary Fig. 8e, 12a). In the tricritical ferroelectric with x_{tcr}^{T}= 0.65 (Fig. 5b), statistical analysis shows that the ferroelastic *c* and *a* domains have different peak values in lattice tetragonality, with $(c/a)_c \approx 1.030$ and $(c/a)_a \approx 1.033$ (Supplementary Fig. 12b). This result is in good agreement with the XRD and SAED results summarized in Fig. 1e. In the composition range of x = 0.50 ~ 0.70, our Monte Carlo simulation results show that the Zr atoms are less active than Ti atoms in generating polar displacement. Furthermore, the tricritical compositional point is found to span in a narrow interval, which supports the scenario shown in Fig. 1e.

Supplementary figures and captions

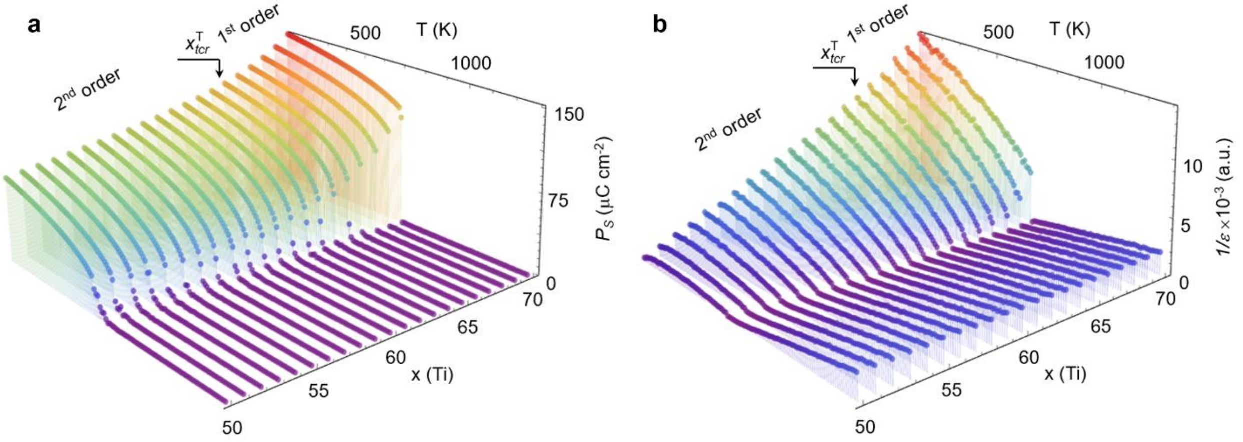

Supplementary Fig. 1. Tricritical point determined by effective Hamiltonian Monte Carlo simulations in the range of 0.50 ≤ x ≤ 0.70. a,**b**, Temperature dependent spontaneous polarization and inverse dielectric constant for the PZT supercells, respectively. Since the experimental results are obtained from un-poled samples, the dielectric response tensor was averaged over all possible field directions to get the numerical results of ε.

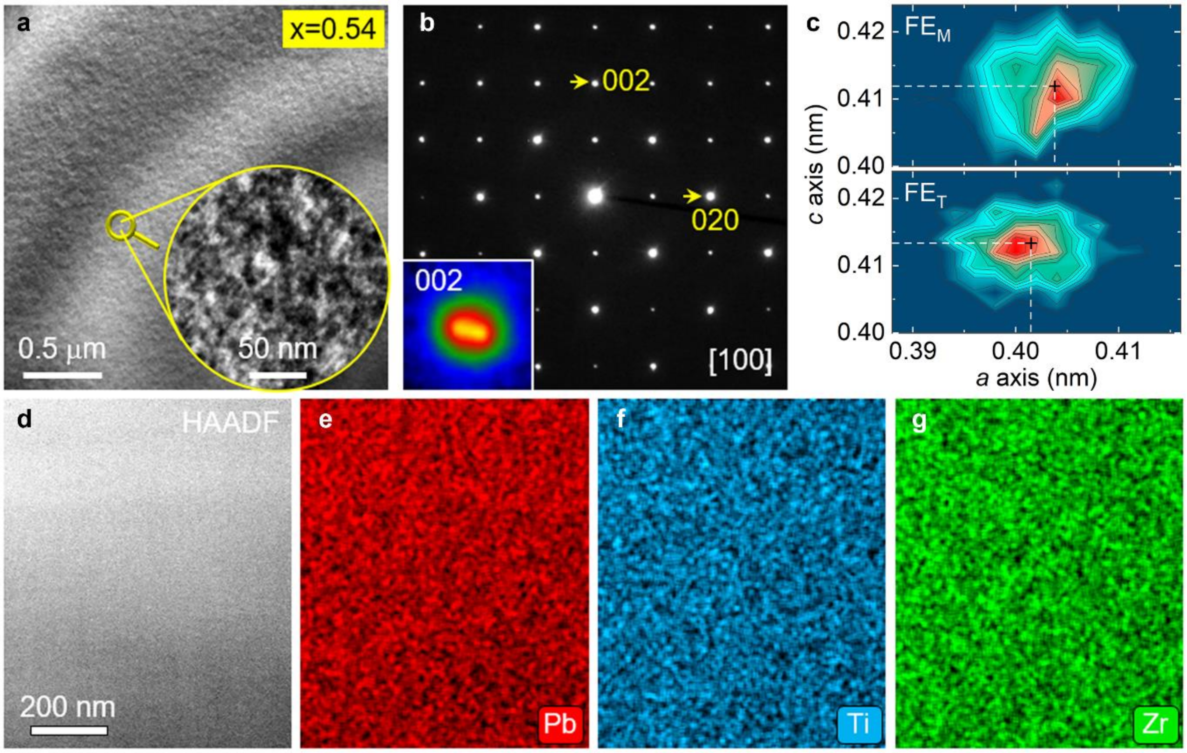

Supplementary Fig. 2. Domain structures in x = 0.54 PZT crystal. a,**b**, Bright-field TEM image and corresponding SAED pattern taken along $[100]_T$ direction. The inset in (**a**) and (**b**) are a two-beam dark-field TEM image and magnified (002) reflection (log plot), respectively. The SAED pattern is indexed based on the pseudocubic unit cell. **c**, 2D lattice parameter plot extracted from a high-resolution TEM image. **d-g**, HAADF-STEM image collected along $[100]_T$ direction and corresponding elemental maps of Pb, Ti and Zr, respectively.

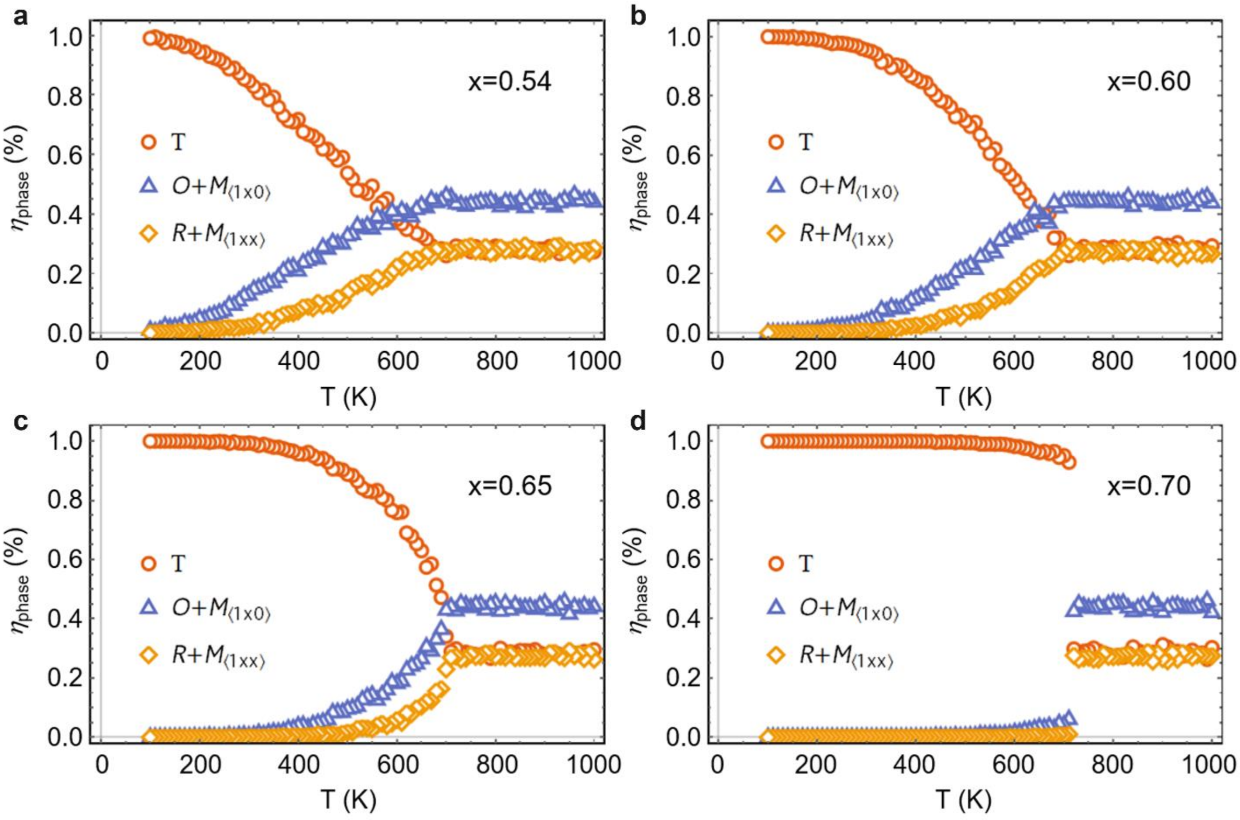

Supplementary Fig. 3. Temperature dependent phase volume fraction obtained by Monte Carlo simulations. a-d, The volume fraction of different structural phases in x = 0.54, 0.60, 0.65 and 0.70 PZT, respectively. Here we considered T-tetragonal, O-orthorhombic, R-rhombohedral and M-monoclinic phases. The subscripts <1x0> and <11x> denote the pseudo-cubic polarization orientation within the monoclinic phases.

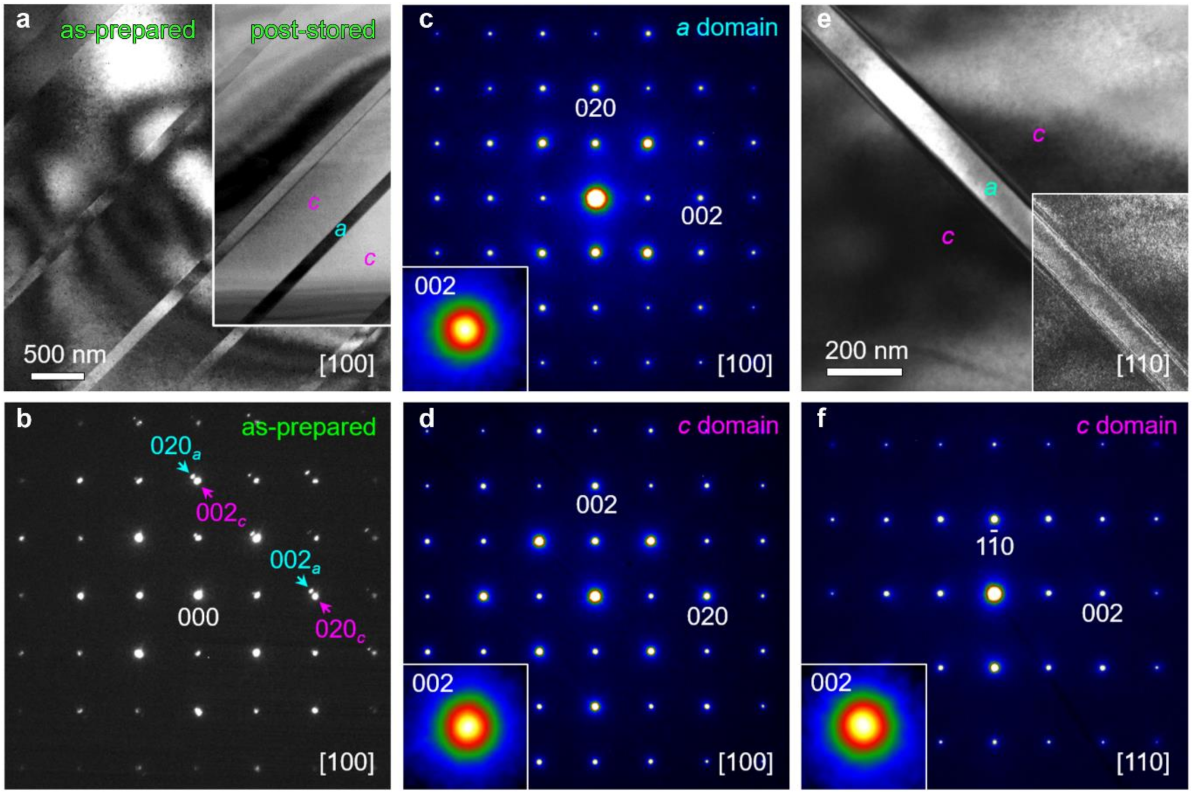

Supplementary Fig. 4. Degeneracy of phase tetragonality in individual ferroelastic domains of $\boldsymbol{x_{tcr}^{\mathrm{T}}}$ = 0.65 crystal. a,**b**, Bright-field TEM image of as-prepared TEM lamella specimen and SAED pattern taken along $[100]_T$ direction. The inset in (**a**) shows the domain morphology after month-level storage. **c**,**d**, SAED patterns of *a* and *c* domains taken from the post-storage lamella specimen. **e**, Bright-field and dark-field (inset) TEM image of $[110]_T$-oriented specimen after storage. **f**, Corresponding SAED pattern taken from the *c* domain. The inset in (**c**, **d**, **f**) shows the magnified (002) reflection with log plot of the intensity distribution, respectively.

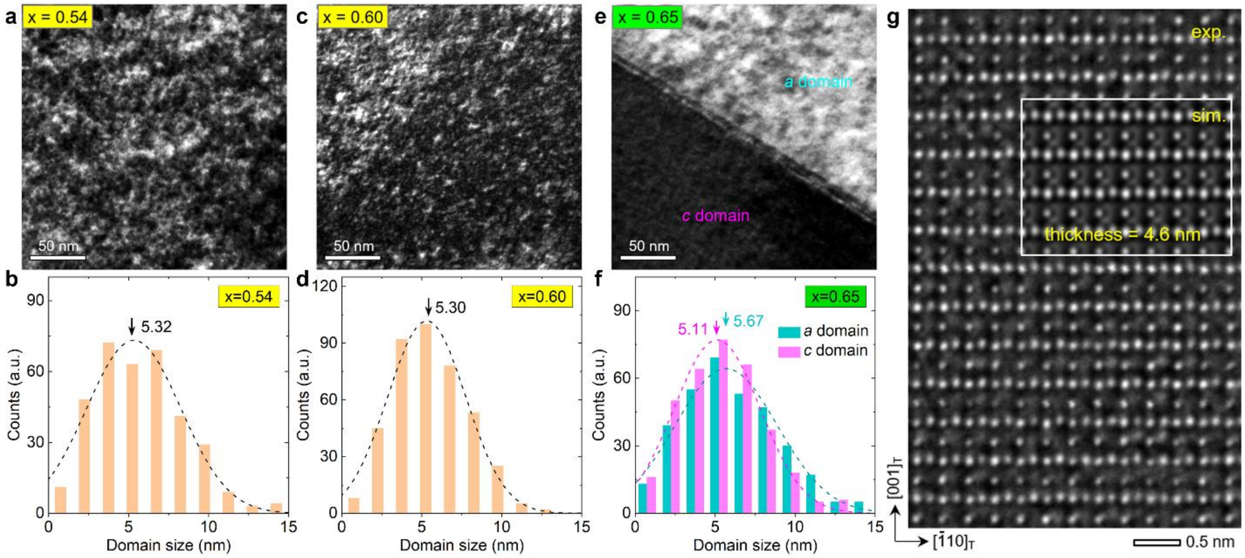

Supplementary Fig. 5. Domain size statistics and representative specimen thickness for NCSI imaging. a-d, Dark-field TEM image taken using **g** = $(020)_T/(002)_T$ reflection from $[100]_T$-/$[110]_T$-oriented PZT (x = 0.54/0.60) crystals and domain size distribution, respectively. **e**,**f**, Dark-field TEM image taken using **g** = $(020)_T$ reflection from $[100]_T$-oriented PZT (x = 0.65) crystal and domain size distribution in ferroelastic *c* and *a* domain, respectively. The dashed lines are Gaussian fitting to the histograms. **g**, Comparison of experimental and simulated (inset) atomic-resolution images recorded along $[110]_T$ direction. The best-fit imaging conditions are *Cs* = -12 μm, *A1* = 1.7 nm, *A2* = 75 nm, *B2* = 50 nm for a specimen with thickness *t* = 4.6 nm and defocus *df* = 6 nm.

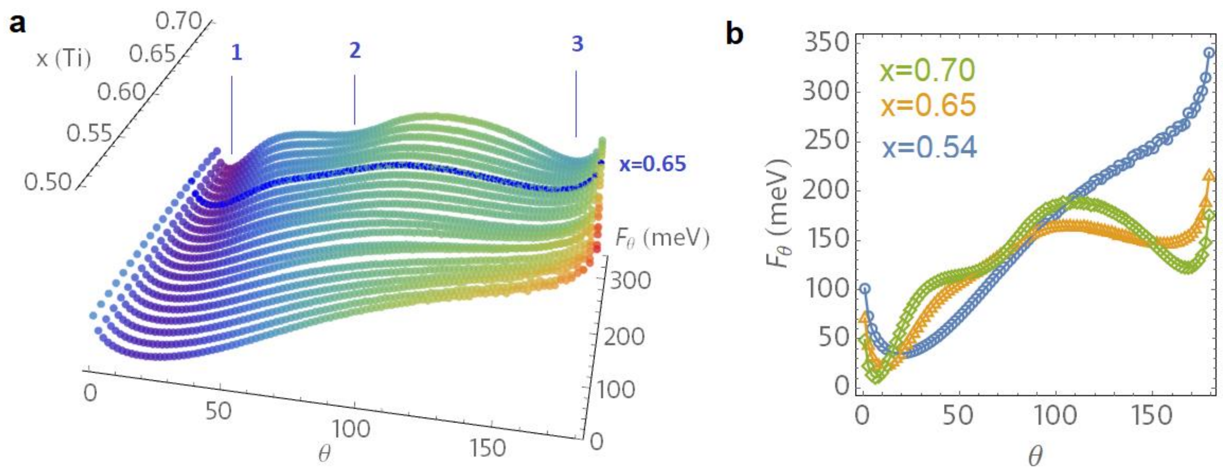

Supplementary Fig. 6. Calculated free-energy F_θ dependence on the angle θ between nearest-neighbor dipoles and various Ti composition x at room temperature. a, The F_θ curves for x ranging from 0.5 to 0.7 with a step of 0.01. The labels 1, 2 and 3 indicate special points of F_θ in the $x > x_{tcr}^{\mathrm{T}}$ region, while the tricritical composition x = 0.65 free-energy curve is highlighted in blue. **b**, Characteristic F_θ behaviors at x = 0.54, 0.65 and 0.7.

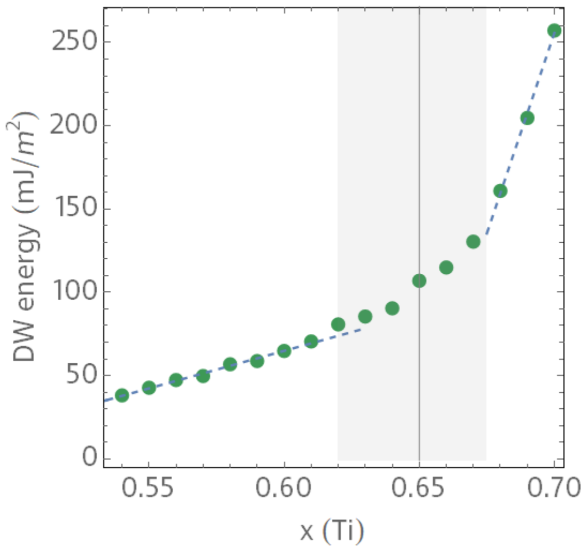

Supplementary Fig. 7. Calculated 90º domain wall energy density as a function of Ti concentration. The domain wall energy monotonically increases with increasing x and features a change of behavior in the vicinity (x region highlighted in gray) of tricritical composition x = 0.65.

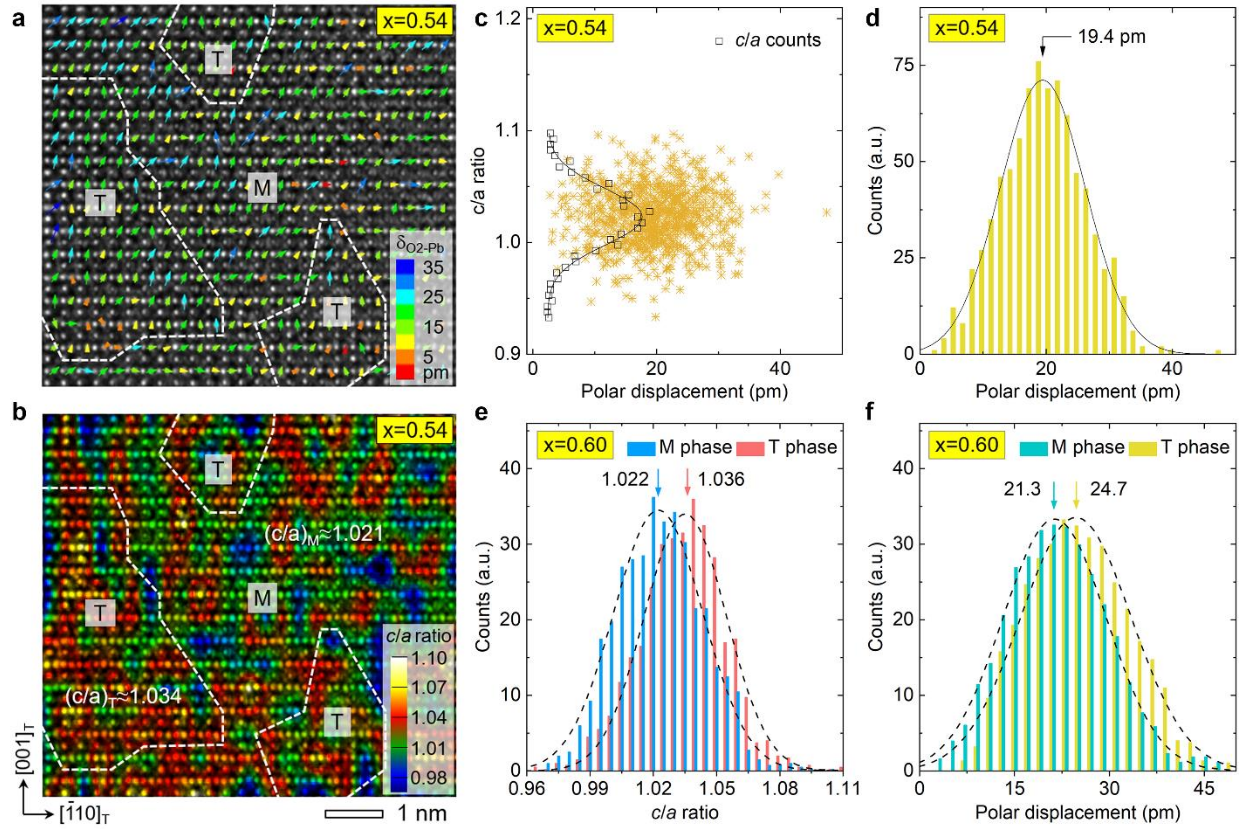

Supplementary Fig. 8. Polarization-strain coupling relation in x < x_{tcr}^{T} PZT crystals. a,**b**, Atomic-resolution TEM image of x = 0.54 PZT crystal recorded along the $[110]_{\mathrm{T}}$ direction with overlapping of (**a**) polar displacement and (**b**) interpolated *c*/*a* ratio map, respectively. **c**,**d**, Unit-cell-wise correlation of *c*/*a* ratio with polar displacement for x = 0.54 crystal and corresponding statistical histogram of polar displacement. The inset in (**c**) shows the statistical profile of corresponding *c*/*a* ratio peaked at 1.024 with FWHM = 0.060. **e**,**f**, Statistical histograms of *c*/*a* ratio and polar displacement for tetragonal and monoclinic phases in x = 0.60 crystal from Fig. 2a. The solid and dashed lines in (**c**-**f**) are Gaussian fitting to the profile and histograms. The local phase overlapping is manifested by elongation of atomic columns along certain direction.

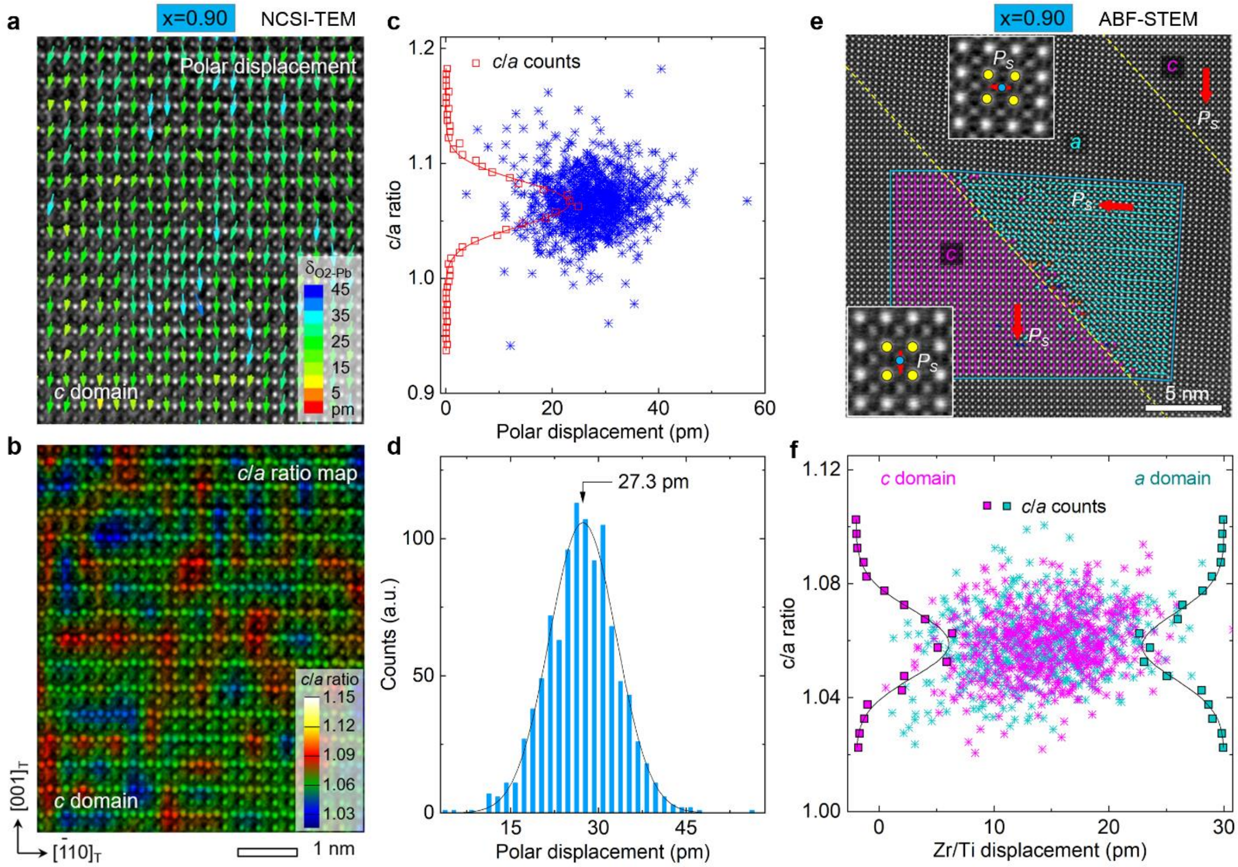

Supplementary Fig. 9. Polarization-strain coupling relation in x > $x_{tcr}^{\mathbf{T}}$ PZT (x = 0.90) crystal. **a**,**b**, Atomic-resolution TEM image recorded along [110]$_T$ direction with overlapping of polar displacement $\delta_{O2\text{-}Pb}$ and interpolated *c/a* ratio map, respectively. **c**,**d**, Unit-cell-wise correlation of *c*/*a* ratio with polar displacement and corresponding statistical histogram of polar displacement. The inset in (**c**) shows the statistical profile of corresponding *c*/*a* ratio peaked at 1.063 with FWHM = 0.046. **e**, ABF-STEM image (reversed contrast) of x = 0.90 PZT collected along [100]$_T$ direction with overlapping of partially mapped Zr/Ti displacements ($\delta_{Zr/Ti\text{-}Pb}$) and enlarged ferroelastic unit cells (Pb-yellow, Zr/Ti-blue). The yellow dashed lines denote positions of the ferroelastic walls. **f**, Unit-cell-wise correlation of *c*/*a* ratio with Zr/Ti polar displacement (peaked at ~14.2 pm) and corresponding statistical profiles of *c*/*a* ratio for *c* and *a* domains peaked at 1.059 with FWHM ≈ 0.028. The solid and dashed lines in (**c, d, f**) are Gaussian fitting to the profiles and histograms.

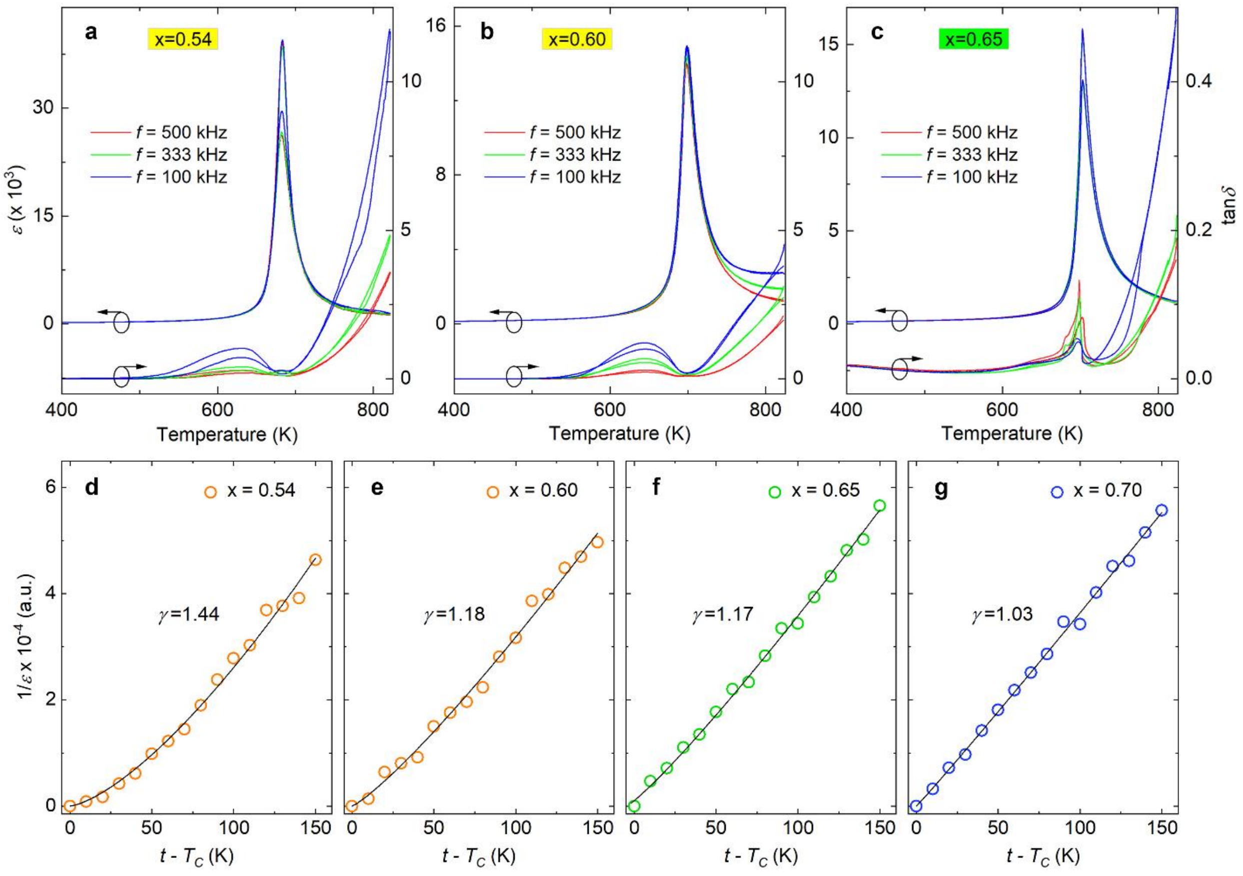

Supplementary Fig. 10. **Dielectric data of PZT crystals and γ exponent from Monte Carlo simulated dielectric constant**. **a-c**, Temperature dependent dielectric constant and loss tangent for x = 0.54, 0.60 and 0.65 crystal measured at a frequency of *f* = 500, 333 and 100 kHz, respectively. **d-g**, Monte Carlo simulated dielectric constant of x = 0.54, 0.60, 0.65 and 0.70 PZT and their fitting (solid lines) using the modified Curie-Weiss law, $1/\varepsilon - 1/\varepsilon_{max} = (t-T_0)^{\gamma}/C$.

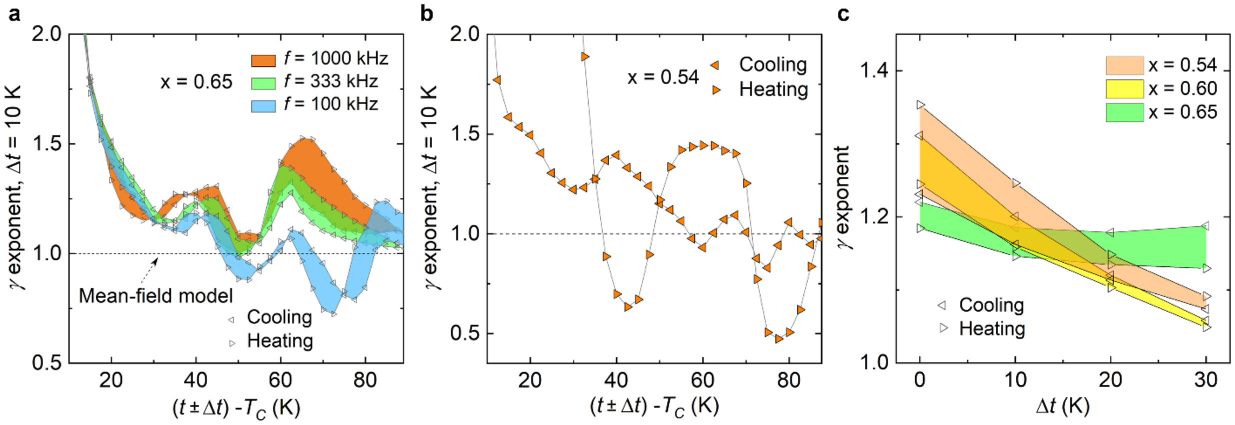

Supplementary Fig. 11. **Temperature and frequency dependent precursor effects. a**, Evolution of dynamic γ exponent for the tricritical ferroelectric measured at f = 1000, 333 and 100 kHz, respectively. **b**, The γ exponent for x = 0.54 crystal measured at f = 500 kHz. The dashed line denotes the mean-field value of $\gamma = 1$. **c**, Changes of γ exponent fitted from the $1/\varepsilon$ data with varied starting temperature away from the T_C in the PZT crystals measured at f = 500 kHz.

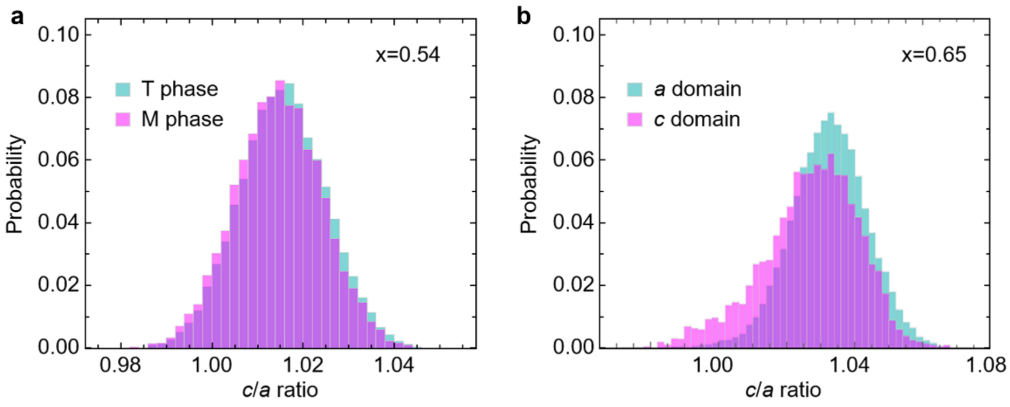

Supplementary Fig. 12. Monte Carlo simulated lattice tetragonality. a, Statistical histograms of *c*/*a* ratio for tetragonal and monoclinic phases corresponding to Fig. 4a in x = 0.54 PZT supercell. **b**, Statistical histograms of *c*/*a* ratio for ferroelastic *c* and *a* domains corresponding to Fig. 4b in x_{tcr}^{T} = 0.65 PZT supercell.

References

1. Rossetti, G. A. & Navrotsky, A. Calorimetric investigation of tricritical behavior in tetragonal $Pb(Zr_xTi_{1-x})O_3$. *J. Solid State Chem.* **144**, 188 (1999).

2. Rossetti, G. A., Khachaturyan, A. G., Akcay, G. & Ni, Y. Ferroelectric solid solutions with morphotropic boundaries: Vanishing polarization anisotropy, adaptive, polar glass, and two-phase states. *J. Appl. Phys.* **103**, 114113 (2008).

3. Chen, Z. *et al.* Giant tuning of ferroelectricity in single crystals by thickness engineering. *Sci. Adv.* **6**, eabc7156 (2020).

4. Prokhorenko, S., Nahas, Y. & Bellaiche, L. Fluctuations and topological defects in proper ferroelectric crystals. *Phys. Rev. Let.t* **118**, 147601 (2017).

5. Boris A. Strukov, Arkadi P. Levanyuk, Ferroelectric Phenomena in Crystals, Springer (Berlin), 1998.

6. Meyer, B. & Vanderbilt, D. Ab initio study of ferroelectric domain walls in $PbTiO_3$. *Phys Rev B* **65**, 104111 (2002).

7. Choudhury, N., Walizer, L., Lisenkov, S. & Bellaiche, L. Geometric frustration in compositionally modulated ferroelectrics. *Nature* **470**, 513-517 (2011).

8. Nahas, Y., Prokhorenko, S. & Bellaiche, L. Frustration and self-ordering of topological defects in ferroelectrics. *Phys. Rev. Lett.* **116**, 117603 (2016).

9. Cohen, R. E. Origin of ferroelectricity in perovskite oxides. *Nature* **358**, 136 (1992).

10. Benedek, N. A. & Birol, T. 'Ferroelectric' metals reexamined: fundamental mechanisms and design considerations for new materials. *J. Mater. Chem. C* **4**, 4000-4015 (2016)
